# Multi-Aspect SAR Target Recognition Based on Prototypical Network with a Small Number of Training Samples

**DOI:** 10.3390/s21134333

**Published:** 2021-06-24

**Authors:** Pengfei Zhao, Lijia Huang, Yu Xin, Jiayi Guo, Zongxu Pan

**Affiliations:** 1 Aerospace Information Research Institute, Chinese Academy of Sciences, Beijing 100194, China; zhaopengfei18@mails.ucas.edu.cn (P.Z.); guojy@aircas.ac.cn (J.G.); zxpan@mail.ie.ac.cn (Z.P.); 2 University of Chinese Academy of Sciences, Beijing 100190, China; 3 Key Laboratory of Technology in Geo-Spatial Information Processing and Application System Chinese Academy of Sciences, Beijing 100194, China; 4 Beijing Institute of Remote Sensing Information, Beijing 100192, China; autoxinyu0624@163.com

**Keywords:** synthetic aperture radar (SAR), automatic target recognition (ATR), multi-aspect SAR, prototypical network, small number of training sample

## Abstract

At present, synthetic aperture radar (SAR) automatic target recognition (ATR) has been deeply researched and widely used in military and civilian fields. SAR images are very sensitive to the azimuth aspect of the imaging geomety; the same target at different aspects differs greatly. Thus, the multi-aspect SAR image sequence contains more information for classification and recognition, which requires the reliable and robust multi-aspect target recognition method. Nowadays, SAR target recognition methods are mostly based on deep learning. However, the SAR dataset is usually expensive to obtain, especially for a certain target. It is difficult to obtain enough samples for deep learning model training. This paper proposes a multi-aspect SAR target recognition method based on a prototypical network. Furthermore, methods such as multi-task learning and multi-level feature fusion are also introduced to enhance the recognition accuracy under the case of a small number of training samples. The experiments by using the MSTAR dataset have proven that the recognition accuracy of our method can be close to the accruacy level by all samples and our method can be applied to other feather extraction models to deal with small sample learning problems.

## 1. Introduction

SAR (synthetic aperture radar) is an active microwave remote sensing device that images an object by transmitting electromagnetic waves and receiving corresponding echoes from the object [1]. SAR can work at night and in bad weather conditions, and can also penetrate the shallow surface and detect concealed targets. It has all-day and all-weather working capability [2]. After decades of development, SAR has become an important method in remote sensing technologies, and it is widely used in military and civilian fields [3].

SAR images are quite different from optical images, due to the mechanism of electromagnetic scattering and coherent imaging. SAR images are the mapping of the three-dimensional geometry and radiation information of the target into the two-dimensional images, which have overlap and shadow, and contain coherent speckle noise. This makes SAR images more difficult to interpret and understand visually than optical images, and thus has a greater impact on target detection and recognition. Therefore, SAR automatic target recognition (SAR ATR) has become a hot research topic.

ATR is used to identify the true attributes of the targets from SAR images. According to the different methods adopted by SAR ATR, the technology can be divided into two classes: classical methods and deep learning methods. Classical methods usually perform feature extraction manually, and then use template matching and machine learning methods for classification, the representative methods of which are given in [4,5,6]. Deep learning methods usually use convolutional neural networks to extract the features of SAR images and then to classify them, the representative methods of which are given in [7,8,9]. Comparing the two types of methods, because of powerful representation capabilities, the ATR methods based on deep learning often have stronger recognition performance. These methods have achieved some good results in target recognition based on single-angle SAR images.

In actual conditions, the performance of the SAR device will be affected by many factors, such as the attitude of platform, the parameters of the radar system, the environment, and the attitude angle of target, which will cause fluctuations to SAR image quality [10]. Due to the side-view imaging geometry, SAR is very sensitive to elevation and azimuth angles of imaging [11]. For the same target, even if the observation angle has a small change, the obtained SAR image of the target may be quite different. Meanwhile, for different targets under a certain observation angle, there may be greater similarity. This makes the target recognition of single-aspect SAR images quite difficult.

**Figure 1:**
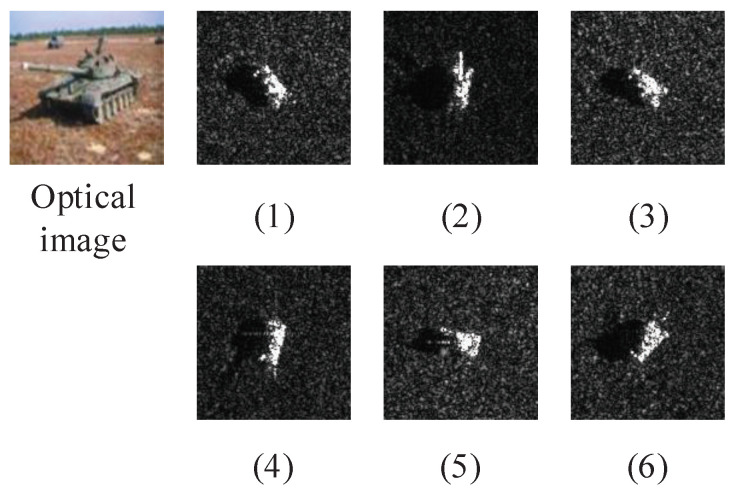
Comparison of the same target’s SAR images in different azimuth angles. Sub-figures (1)–(6) are SAR images of T72 tank in different azimuth angles.

Figure 1 shows the images of the airborne SAR in MSTAR (Moving and Stationary Target Acquisition and Recognition) dataset [12], observing the same target (T72 tank) at different azimuth angles. Figure 1 (2),(6) can clearly show the barrel of T72, while other images are not observed or not obvious. Therefore, the single-aspect SAR target recognition method may fluctuate in performance as the azimuth angle changes, and it is often difficult to achieve the requirements of high-precision recognition.

With the development of SAR system technology, advanced SAR can continuously observe the same target at different azimuth angles. The SAR images of the same target at multiple azimuth angles have more information for identification [13]. Multi-aspect SAR target recognition uses the image sequence of the same target at different azimuth angles, where the scattering characteristics of different angles are helpful for target recognition. Compared with single-aspect target recognition, it has some advantages [14,15]:

1. SAR image sequence with multiple azimuth angles can make up for the lack of information in a single image, and further increase the information for target recognition.
2. There is a certain correlation involved in multi-aspect SAR images, which provides another dimensional of information for target recognition.

In recent years, with the development of deep learning, multi-aspect SAR target recognition methods based on deep learning have also been developed. Pei et al. proposed the multi-view deep convolutional neural network (MVDCNN), which uses a parallel network to extract features from SAR images of different views, and further fuses these features through pooling [16]. Zhao et al. proposed the multi-stream convolutional neural network (MS-CNN) and designed a Fourier feature fusion framework derived from kernel approximation based on random Fourier features to unravel the highly nonlinear relationship between images and classes [17]. Zhang et al. proposed a multi-aspect ATR architecture based on ResNet and LSTM, which achieves good recognition performance [18].

Although these methods have provided satisfactory results, they all require large-scale training data to train the deep learning model. Once the size of the dataset is reduced, the performance of target recognition will also decrease.

In addition, the SAR dataset of a specific target in a different azimuth angle is usually expensive to obtain and requires a lot of manpower and material resources to label sufficient samples for image annotation. Therefore, multi-aspect SAR target recognition under a small number of samples is the current development trend.

At present, single-apect SAR target recognition under the small number of training samples has been studied [19,20,21]. However, there are relatively few studies on multi-apect SAR target recognition with a small number of training samples. Therefore, this paper proposes a recognition method based on a prototypical network [22], which uses a deep learning model as a feature extraction module, and further uses methods such as multi-task learning and multi-level feature fusion based on attention mechanism.

The advantages of the method in this paper are as follows:

1. Experiments show that this method can significantly improve the recognition performance of the model with a small number of training samples.
2. This method can be applied to different deep learning feature extraction models.

The remainder of the paper is organized as follows. Section 2 introduces the target recognition method under the small number of training samples proposed in this paper in detail; Section 3 presents the experimental details and results; Section 4 discusses the advantages and generality of the method in this paper; Section 5 summarizes the full paper.

## 2. Methods

### 2.1. Prototypical Network

Prototypical network [22] is a classical method for few-shot learning. The basic idea of the prototypical network is: For classification tasks, the prototypical network maps the samples of each class to a specific space, and uses the center point of each class in the specific space as the prototype of the class. The prototypical network uses the Euclidean distance to measure the distance between the sample and the prototype. During the training process, the sample is gathered in a specific mapping space, so that it is close to the prototype of its own class and far away from other prototypes. During the testing process, the Euclidean distance in the feature space from each test sample to each prototype is calculated, and the softmax classifier is used to classify the test samples according to the distance.

This paper has made some modifications to the prototypical network method, in which the classes of training set and test set are consistent.

**Figure 2:**
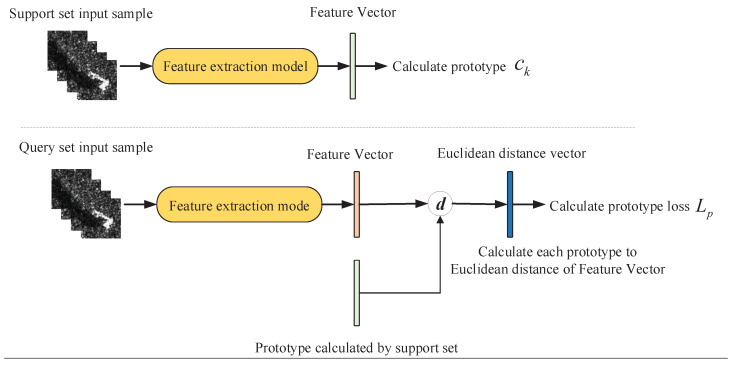
Schematic diagram of method training.

The training of the prototypical network method is shown in Figure 2. There are *N* classes of training set and test set. During training, each training episode randomly puts forward $K_{S}$ samples from each of *N* classes of training set as support set $S=\{\left(x_{1},y_{1}\right),\left(x_{2},y_{2}\right),\ldots ,\left(x_{N*K_{S}},y_{N*K_{S}}\right)\}$, as well as randomly putting forward $K_{Q}$ samples as query set $Q=\{\left(x_{1},y_{1}\right),\left(x_{2},y_{2}\right),\ldots ,\left(x_{N*K_{Q}},y_{N*K_{Q}}\right)\}$, where $x_{i}$ is the multi-aspect SAR images and $y_{i}$ is the sample label. Through the deep learning feature extract model $f_{\varphi }:R^{D}\to R^{M}$, each D-dimensional training sample is mapped to a high-dimensional space of dimension M, and $c_{k}$ is calculated by $S_{K}$, where $S_{K}$ is the support set of class *k*, and $c_{k}$ is the prototype of class *k*, $c_{k}$ is obtained by taking the average value of all samples in $S_{K}$ in the high-dimensional space, as in Formula (1).

(1) $$c_{k}=\frac{1}{S_{K}}\sum_{\left(x_{i},y_{i}\right)\in S_{K}}f_{\varphi }\left(x_{i}\right)$$

After calculating the prototype, the prototypical network uses the Softmax function to calculate the distance between the samples in the query set *Q* and each prototype, as shown in Formula (2), where it is the Euclidean distance, as shown in Formula (3).

(2) $$p_{\varphi }\left(y=k|x\right)=\frac{exp(-d\left(f_{\varphi }\left(x\right),c_{k}\right))}{\sum_{k}exp\left(-d\left(f_{\varphi }\left(x\right),c_{k}\right)\right)}$$

(3) $$d\left(X,Y\right)=\sqrt{\sum_{i=1}^{n}\left(x_{i}-y_{i}\right)}$$

The deep learning model is optimized by reducing the distance between the sample in the query set *Q* and the center of the class prototype. The training loss is shown in Formula (4), where *k* is the true label of training sample.

(4) $$L_{p}=-\log p_{\varphi }\left(y=k|x\right)$$

In the testing process, first we use all the training set samples to calculate the prototype of each class, and then calculate the distance from each test sample to each prototype through Formula (2). The test sample is divided into the class with the closest prototype.

### 2.2. Multi-Task Learning

**Figure 3:**
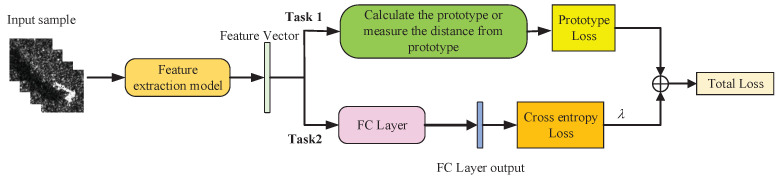
Multi-task learning model.

The prototypical network method proposed in Section 2.1 can measure the distance between the test sample and the center of prototype, but cannot directly classify a single sample. Therefore, we try to add a fully connected layer after the deep learning model to classify the samples on the basis of the prototypical network method, and construct an additional classification task based on cross-entropy loss to form a multi-task learning model, as shown in Figure 3.

Through the learning model shown in Figure 3, the distance measurement task and the classification task of the prototypical network can assist each other, so that the model can learn effective information from the classification task that is helpful for the distance measurement task, and further improve the performance of prototypical network.

In addition, since each task has a different noise mode, the measurement task and classification task at the same time can average the impact of noise and share the overfitting risk of each task, thus improving the generalization performance of the model and reducing the impact of SAR image speckle noise on classification [19].

The multi-task learning model includes a distance measurement task of prototypical network and a classification task. The classification task uses cross-entropy loss to optimize the model. Therefore, the joint loss of the model is shown in Formula (5), which is divided into two parts. *L* is total loss, $L_{CE}$ represents the cross-entropy loss, $L_{P}$ is the distance metric loss, and λ is the hyperparameter.

(5) $$L=L_{P}+\lambda *L_{CE}$$

### 2.3. Feature Extraction Model

The prototypical network method requires a feature extraction model to map the sample to a specific space. This paper uses a multi-channel convolutional neural network as a feature extraction model. That is, we use multiple weight-sharing CNNs to extract the features of a single SAR image, and then use the fully connected layer to classify the features of multiple SAR images.

This paper mainly inroduces the NLECA-EfficientNet model into the feature extraction model for experiments. The model uses multi-channel EfficientNet-B0 [23] as the backbone network, and adds the NLECA channel attention module to recalibrate the multi-aspect SAR image features. It can enhance the more useful features for classification, so as to achieve high accuracy for multi-aspect SAR target recognition. In addition, this paper also uses multi-channel ResNet [24], VGGNet [25] and AlexNet [26] as feature extraction models in experiments (Section 4) to verify the generality of the method in this paper.

#### 2.3.1. EfficientNet

The EfficientNet series model is one of the deep learning classification models with the best classification performance at present, and its basic network is obtained through neural network architecture search technology [23]. In this paper, EfficientNet-B0 is selected. This model has the least parameters and the fastest inference speed among the EfficientNet series models. It is more suitable for small-scale datasets such as the SAR dataset. The structure of EfficientNet-B0 is shown in Table 1,where *k* is the number of classes.

#### 2.3.2. NLECA Module

NLECA (Non-Local Efficient Channel Attention) module is a channel attention module improved from ECA (Efficient Convolution Attention) module [27].

**Figure 4:**
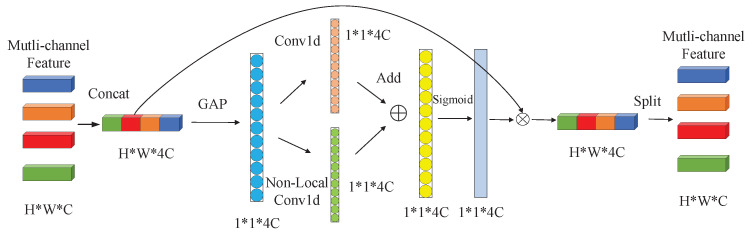
Schematic diagram of NLECA module.

The ECA module captures the local cross-channel interaction by considering each channel and its *k* neighboring channels, this module implements the above operations through a one-dimensional convolution with the convolution kernel of *k*. If the ECA module is directly added to the multi-channel CNN for multi-aspect SAR target recognition tasks, the current feature channel can only perform cross-channel information interaction with nearby *k* feature channels, and these feature channels usually come from the same SAR target image, which cannot realize information interaction among multi-aspect SAR images. For example, Figure 4 is a schematic diagram of the NLECA module with the number of input multi-aspect images is 4, the image features from 4 CNNs are concentrated and pooled to obtain a feature vector with a dimension of 1∗4C, where *C* is the number of feature channels and is usually much larger than the size of the convolution kernel *k*. If only one-dimensional convolution is performed, the current *k* convolution values usually come from the same input image. Therefore, the use of one-dimensional convolution cannot realize information interaction among different input images, and thus cannot make full use of multi-aspect SAR information.

**Figure 5:**
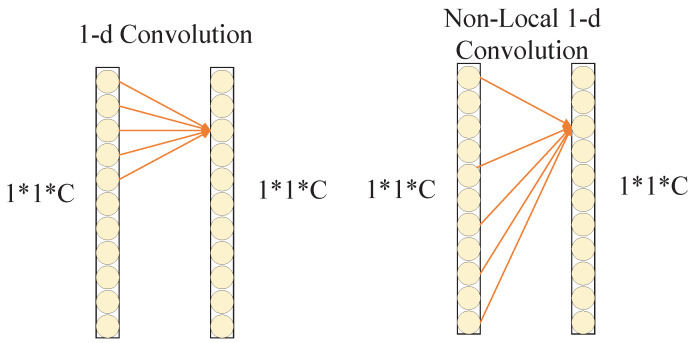
Conv1d and Non-Local Conv1d.

The NLECA module adopts non-local one-dimensional convolution (Non-Local Conv1d) to obtain non-local information. Non-local one-dimensional convolution is an one-dimensional implementation of Deformable Convolution Net [28]. The diagram of one-dimensional convolution is shown on the left side of Figure 5. If the size of the convolution kernel is *k*, it can only perform convolution operations on local *k* values. Instead of non-local one-dimensional convolution, any *k* values in the vector can be selected for convolution operation by learning, which makes up for the shortcomings of the ECA module and realizes the feature information interaction among multi-aspect SAR images.

**Figure 6:**
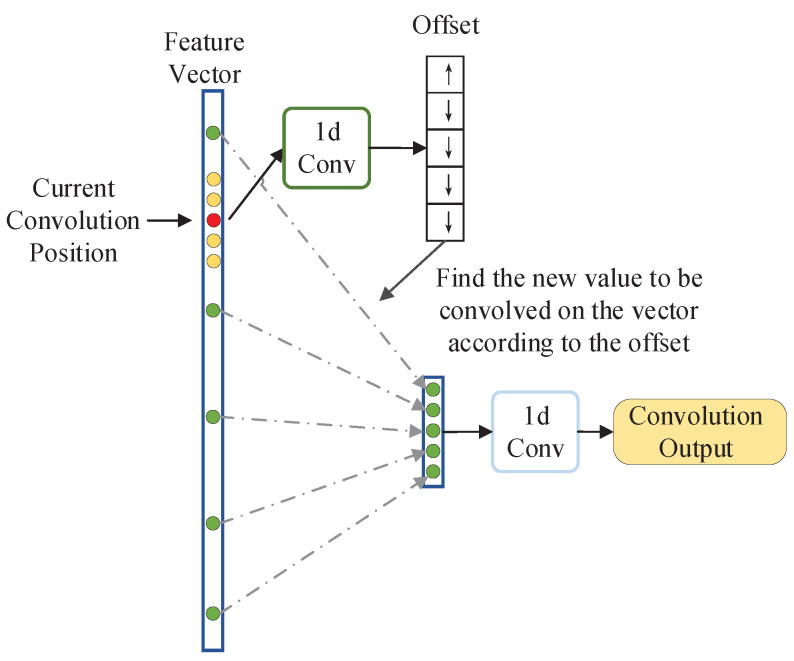
Non-local one-dimensional convolution structure diagram.

The structure of non-local one-dimensional convolution is shown in Figure 6. It first obtains a set of offsets of the current convolution position through a one-dimensional convolution operation which kernel size is *k*, and then uses this set of offsets to find *k* non-local convolution values, thus realizing a non-local one-dimensional convolution operation.

About the calculation process of the NLECA module, first, the features of size H×W×C obtained by 4 CNN channels are concatenated into feature *F* of size H×W×4C. Then, the global average pooling on the feature *F* is performed to obtain a global feature vector *F*, and one-dimensional convolution and non-local one-dimensional convolution on the feature vector *V* is performed to obtain two weight vectors $W_{1}$ and $W_{2}$. The two weight vectors are added and normalized by the Sigmoid function to obtain the final weight vector *W*. After that, the feature *F* and the weight vector *W* are dotted to calculate the output feature $F_{out}$. Finally, $F_{out}$ is split into feature vectors of size H×W×C and sent back to the multi-channel CNN.

Through the combination of one-dimensional convolution and non-local one-dimensional convolution, the NLECA module not only has the ability of ECA module to improve classification performance from local channel information, but also has the ability of non-local information interaction. When NLECA module is inserted into a multi-channel CNN for multi-aspect SAR image recognition tasks, information interaction among multi-aspect SAR images can be further realized, thereby improving the recognition performance.

#### 2.3.3. NLECA-EfficinentNet

**Figure 7:**
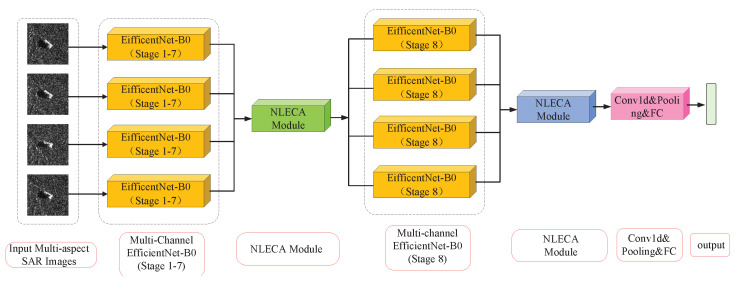
Multi-channel EfficientNet-B0 embedded with NLECA module.

The NLECA-EfficienctNet model used in the experiment is shown in Figure 7. The NLECA module is inserted before and after the last MBConv stage of EfficientNet-B0 at the same time. The model uses the channel attention mechanism to re-calibrate the features of the multi-channel image, by enhancing the information useful for classification among the global information, as well as suppressing the information useless for classification, so as to achieve high accuracy in multi-aspect SAR target recognition. The recognition performance of the method under the MSTAR dataset is shown in Section 3.

When used as a feature extraction model, this paper removes the fully connected layer part of the model.

### 2.4. Multi-Level Feature Fusion

In deep convolutional neural networks, low-level image features have high resolution and more detailed information, but low-level image features are more noisy and have poor semantics; while high-level image features have higher-level semantics, but its resolution is lower, the detailed information are less, and the high-level features are more prone to over-fitting; the middle-level image features are in-between the high-level and low-level features [20,21]. Effective fusion of these three features can improve the classification performance and make the model more robust.

This paper uses multi-level feature fusion to improve the accuracy of multi-aspect SAR target recognition under a small number of samples. Thus, the low-, medium- and high- levels image features of the multi-aspect SAR images are integrated to classify.

**Figure 8:**
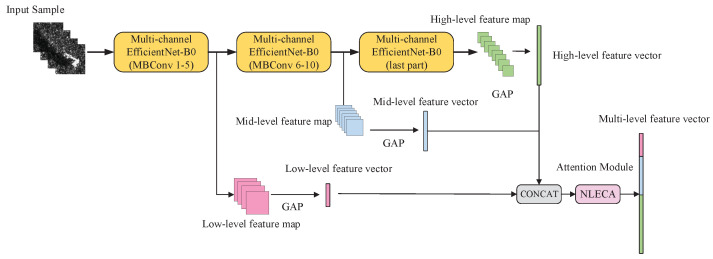
Schematic diagram of multi-level feature extraction and fusion.

As shown in Figure 8, NLECA-EfficientNet is used as a feature extraction model, and its backbone network is EfficientNet-B0. The feature maps output from the 5th MBConv Stage, the 10th MBConv Stage, and the last layer of EfficientNet-B0 are low-, middle-, and high-level features separately. The dimensions of the low-level, middle-level, and high-level feature maps, which are given by $F_{L}$, $F_{M}$, and $F_{H}$, are $\left(N,H_{L},W_{L},C_{L}\right)$, $\left(N,H_{M},W_{M},C_{M}\right)$, and $\left(N,H_{H},W_{H},C_{H}\right)$, respectively. *N*, *H*, *W*, and *C* are the batch sizes of the input samples, the height, the width, and the channel number of the feature map. However, due to the different heights, widths, and channel numbers of low, medium, and high-level feature maps, they cannot be directly concatenated.Therefore, we perform global average pooling on $F_{L}$, $F_{M}$, and $F_{H}$ to obtain feature vectors $V_{L}$, $V_{M}$ and $V_{H}$ with dimensions $\left(N,C_{L}\right)$,$\left(N,C_{M}\right)$ and $\left(N,C_{H}\right)$ respectively. Then we concatenate the feature vectors to obtain a vector *V* whose dimension is (N,C), $C=C_{L}+C_{N}+C_{H}$. The calculation process is given in Formulas (6) and (7), where GAP means global average pooling, and CONCAT means vector concatenation.

(6) $$\left\{\begin{array}{cc} V_{L} & \mathrm{GAP}(F_{L})\\ V_{M} & \mathrm{GAP}(F_{M})\\ V_{H} & \mathrm{GAP}(F_{H}) \end{array}\right.$$

(7) $$V=\mathrm{CONCAT}(V_{L},V_{M},V_{H})$$

It should be noted that the vector *V* contains not only the features of multi-aspect SAR images, but also the three-level features of high-, medium- and low- levels. These types of features have different effects on classification results, so we use the NLECA module to recalibrate the vector *V* to enhance the useful features, and thus obtain the final output $V_{out}$.

After passing through the NLECA module, $V_{out}$ is further used for direct classification, calculation, and measurement of the prototype. The operation of the attention mechanism here not only realizes the information interaction of low-, medium-, and high-levels, but also realizes the information interaction among multi-aspect SAR images, which makes full use of the feature information and thus improves the performance and the generality of the model.

### 2.5. Training and Testing Tricks

In addition, this paper also uses some tricks to improve the performance of multi-aspect SAR ATR under the small number of training samples.

#### 2.5.1. Image Preprocessing

Since the SAR imaging angle of target has a great influence on the image feature, it is not proper to use large-angle rotation, mirroring and other optical image augmentation methods to augment the SAR samples. With reference to the method in [19], after constructing a multi-aspect SAR images dataset, each image of the training set is rotated by $\pm 10^{{}^{\circ}}$, hereby increasing the training set by three times. In addition, for coherent speckle noise reduction, this paper also uses the refined Lee filter [29] for preprocessing.

#### 2.5.2. Label Smoothing

Label smoothing is a regularization method in machine learning. It changes the one hot encoding of the label vector *y* into soft one-hot encoding, thereby adding some noise to the label, reducing the weight of the correct class and the overfitting problem of the model. After using label smoothing, the classification loss changes from cross-entropy $L_{CE}$ to $L_{B}$, as shown in Formula (8), where ε is a small hyperparameter, the value of ε is usually 0.1, and target represents the class of current samples [30].

(8) $$L_{B}=\left\{\begin{array}{c} \left(1-\varepsilon \right)\times L_{CE},\mathrm{if}\left(i=target\right)\\ \varepsilon \times L_{CE},\mathrm{if}\left(i\neq target\right) \end{array}\right.$$

#### 2.5.3. Test Time Augmentation

The same augmentation method for testing dataset, as that for training dataset, can also effectively improve the test accuracy.

Each test sample is first rotated by $\pm 10^{{}^{\circ}}$ to add up one sample to three samples. Then, the three samples are produced by feature extraction model to obtain three corresponding feature vectors. These three vectors are averaged to obtain a new vector, which is then used to measure the distance from test sample to the prototype.

This method is similar to the bagging method in machine learning. It can effectively generalize the model.

### 2.6. Multi-Aspect SAR ATR Method with Small Number of Training Samples

**Figure 9:**
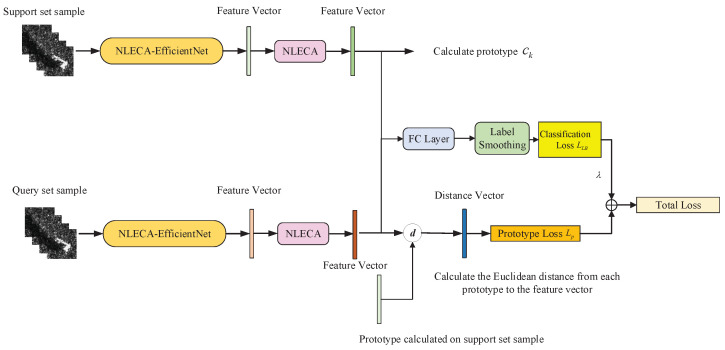
Schematic diagram of model training process.

Combining the above methods and using NLECA-EfficientNet as the feature extraction model, the multi-aspect SAR ATR training framework proposed in this paper under the small number of samples is shown in Figure 9.

The training process has multiple epochs, and each epoch is divided into multiple episodes, as shown in Figure 9. The training steps for each episode are as follows:

1. Randomly select $K_{S}$ samples from each of the *N* classes in the training set as the support set $S=\{\left(x_{1},y_{1}\right),\left(x_{2},y_{2}\right),\ldots ,\left(x_{N*K_{S}},y_{N*K_{S}}\right)\}$, and select $K_{Q}$ samples as the query set $Q=\{\left(x_{1},y_{1}\right),\left(x_{2},y_{2}\right),\ldots ,\left(x_{N*K_{Q}},y_{N*K_{Q}}\right)\}$, where $x_{i}$ is the multi-aspect SAR images, $y_{i}$ is the sample label, and there are a total of $N\times (K_{S}+K_{Q})$ training samples participate in each training episode.
2. Use the training samples in the support set to calculate the prototype of each class.That is, the support set samples of each class are used to obtain multi-level feature vectors through the multi-aspect SAR feature extraction model, and the multi-level feature vectors of each class are averaged to obtain the prototype.
3. The samples of the query set are used to obtain the feature vector through the feature extraction model, which is used to calculate the Euclidean distance from each prototype. The loss $L_{P}$ is calculated using Formulas (2) and (4).
4. Use the multi-level feature vectors of all samples in the support set and the query set to perform the classification task, and obtain the classification loss $L_{B}$ by Formula (8).
5. Perform a weighted addition on the prototype loss $L_{P}$ and the classification loss $L_{B}$ to get the total loss, while backpropagates to adjust the model parameters.

**Figure 10:**
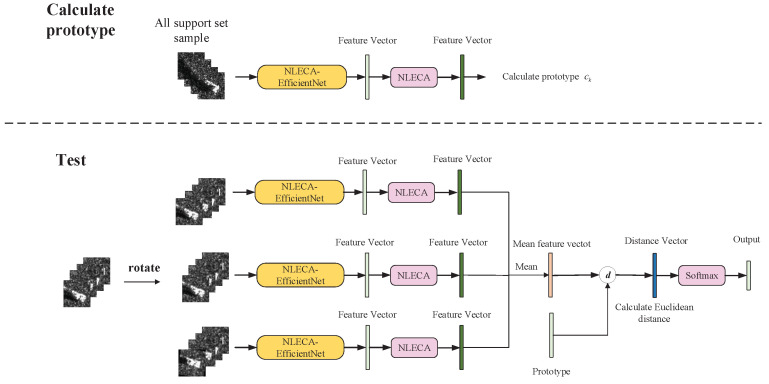
Schematic diagram of model test.

The test method is shown in Figure 10. The test steps for a single sample are as follows:

1. Use the trained feature extraction model and use the entire training set to calculate the prototype of each class.
2. Rotate each image of the test sample by $\pm 10^{{}^{\circ}}$, and extend the test sample by three times, obtain three feature vectors by the trained feature extraction model, and average the three feature vectors to obtain a new feature vector.
3. Use the feature vector in step 2 and the prototype in step 1 to calculate the Euclidean distance and obtain the distance vector, and then use the Softmax classifier to classify the distance vector.

## 3. Experiments and Results

### 3.1. Experimental Dataset

#### 3.1.1. MSTAR Dataset

The moving and stationary target acquisition and recognition (MSTAR) dataset is used in our experiments. The azimuth angle of the target in MSTAR dataset is in the range of 0${}^{{}^{\circ}}$–360${}^{{}^{\circ}}$, so it is suitable as the multi-aspect target recognition dataset [12].

According to the dataset construction proposed in [19], considering the azimuth angle coverage of SAR, we select 4 images withtheazimuth angle variation within 45${}^{{}^{\circ}}$(which can be realized by satellite SAR system) as a set of multi-aspect SAR image sequence.

We constructed 4 datasets, SOC, EOC1, EOC2, and EOC3 dataset. In SOC (Standard operating condition) dataset, the acquirements of training and testing samples by SAR sensors are similar, where the training data is captured at depression angle of 17${}^{{}^{\circ}}$ and the testing data is captured at 15${}^{{}^{\circ}}$, and the number of classes is 10. In EOC (Extended operating condition) datasets, the acquirements of training and testing samples are dissimilar, because the SAR images are very sensitive to depression angle. The training data of EOC1 is captured at depression angle of 17${}^{{}^{\circ}}$ and the testing data is captured at 30${}^{{}^{\circ}}$, and the number of classes is 4. The size of SOC dataset is shown in Table 2 and the size of EOC1 dataset is shown in Table 3.

EOC2 and EOC3 datasets refer to vehicle version changes and vehicle configuration changes, respectively. Version changes refer to the functional changes of the vehicles, that is, the original vehicles changes to ambulances, transport vehicles, reconnaissance vehicles, etc. Configuration changes refer to the addition or removal of some parts of the vehicles, such as the oil tank removal of T72.

In the experiments, BMP2, BRDM_2, BTR70 and T72 in SOC training set are selected as the training set for both EOC2 and EOC3. Five types of version changes of T72, including S7, A32, A62, A63, and A64, are the testing set of EOC2. Two types of configuration changes of BMP2, including 9566 and C21, and five types of configuration changes of T72, including 812, A04, A05, A07, and A10, are the testing set of EOC3 [17].

The total number of training sets of EOC2 and EOC3 is 4473.The total number of testing sets of EOC2 and EOC3 are 9996 and 12,969, respectively.

#### 3.1.2. Small Number of Training Datasets

Based on the dataset on Section 3.1.1, this paper performs uniform under-sampling to obtain some small number of training datasets, which are given as follows:

1. SOC training set: This paper establishes two under-sampled SOC datasets, SOC-150 and SOC-250. SOC-150 has 10 classes with only 15 samples per class, a total of 150 training samples; SOC-250 has 10 classes with only 25 samples per class, a total of 250 training samples. The two datasets only account for 1.42% and 2.36% of the original training set size, respectively.
2. EOC1 training set: After under-sampled from original EOC1 training set, two datasets, EOC1-100 and EOC1-200, are obtained. There are only 25 samples in each of the four classes of EOC1-100, a total of 100 training samples.There are only 50 samples in each of the 4 classes in EOC1-200, a total of 200 training samples. The two datasets account for 2.26% and 4.53% of the original training set size, respectively.
3. EOC2 and EOC3 training sets: After under-sampled from original EOC2 training set (the same as original EOC3 training set), two datasets, EOC23-100 and EOC23-200 are obtained. The EOC23-100 data set has 4 classes with only 25 samples per class, a total of 100 training samples. There are 50 samples in each of the 4 classes in E0C23-200 dataset, a total of 200 training samples. The two datasets account for 2.23% and 4.47% of the original training set size, respectively.

### 3.2. Experimental Environment

All experiments in this paper are conducted under Ubuntu 18.04 system, using the deep learning framework Pytorch 1.6. Intel i9-9900 CPU and NVIDIA RTX 2080Ti GPU are used as the key hardware.

### 3.3. Experimental Parameters

The experiments in this paper are performed in two categories:

1. Use a small number of samples to train and test the model directly. In this part, center loss is used as the training loss [31], and the cosine decay is used as the learning rate reduction strategy. Other parameters are shown in Table 4.
2. Use the method proposed in this paper for training and testing. The Epochs for training are all set to 100, where each epoch trains 200 episodes, the Adam optimizer is used for learning, the learning rate is 1e-3, and the learning rate for each 20 epoch is reduced to 1/2. For SOC-150 training set, the number of support sets $K_{S}$ for each episode is 5, and the number of query sets $K_{Q}$ is 10. For SOC-250 training set, the number of support sets $K_{S}$ is 10, and the number of query sets $K_{Q}$ is 15. For the training sets of the EOC1 (EOC1-100, EOC1-200) and EOC23 (EOC23-100, EOC23-200), the number of support sets $K_{S}$ for each episode is 10, and the number of query sets $K_{Q}$ is 10.

In addition, for the NLECA attention mechanism, the convolution kernel size is 7; the addition ratio of one-dimensional convolution and non-local one-dimensional convolution is 2:1. For the loss function of multi-task learning in Formula (5), the value of λ is set to be 0.2 for experiments.

### 3.4. Results of NLECA-EfficientNet Experiments

We use NLECA-EfficientNet as the feature extraction model. We conduct experiments on small number of training datasets, and compare their results with the experimental results of complete dataset, as shown in Table 5, Table 6, Table 7 and Table 8.

The experimental results of SOC datasets are shown in Table 5. The recognition accuracy improvement of this method on SOC-150 dataset is 21.34%, and that on SOC-250 dataset is 11.32%. This method uses only 2.2% of the complete training dataset to obtain the recognition accuracy of 99.97%, which is only 0.03% behind the recognition accuracy of the complete training dataset.

The experimental results of EOC1 datasets are shown in Table 6. Since this method uses the distance measurement of prototype to classify, the improvement of this method is significantly improved, especially when the EOC1 dataset has only 4 classes. The accuracy improvement of this method on EOC1-100 dataset reaches 29.80%, and that on EOC1-200 dataset is 11.32%.

The experimental results of EOC2 and EOC3 are shown in Table 7 and Table 8. This method has certain improvement in both datasets. In EOC3, the direct training results have high accuracy, so the improvement of this method is relatively limited.

This method has different degrees of improvement on each dataset of MSTAR; moreover, the recognition accuracy of this model in the case of a small number of training samples is very close to the recognition accuracy of the direct training and testing model in case of a complete training dataset.

### 3.5. Results of Ablation Experiments

In order to verify their own improvement of each method proposed above, this section conducts ablation experiments by parts of the methods combination on the SOC-150 training set and SOC-250 training set. The results are shown in Table 9.

As the baseline, we use the NLECA-Efficient model as the feature extraction model, and directly train the model to obtain the experimental results. On this basis, the prototypical network, the multi-task learning method, the multi-feature fusion method, the attention mechanism, the Label Smoothing method, and the test time augmentation are added one by one in sequence. The results correspond to numbers 1–7 are shown in Table 9. Moreover, the time costs are shown in Table 10.

**Figure 11:**
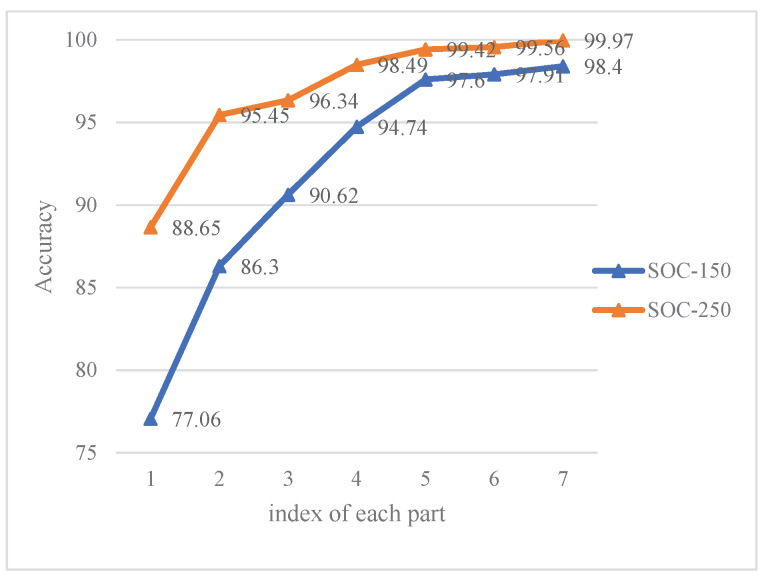
Diagram of recognition accuracy of ablation experiments.

It can be seen from Table 9 and Figure 11 that each method proposed in this paper has a certain effect on improving the performance of target recognition.The use of prototypical network has the greatest improvement in performance, increasing by 9.24% and 6.80% on the SOC-150 and SOC-250 datasets, respectively. Secondly, the multi-feature fusion method also significantly improves the performance, increasing by 4.12% and 2.15% on two datasets respectively. After adding the attention mechanism on the basis of multi-feature fusion, the performance has been further improved, and the two datasets have been improved by 2.86% and 0.93% respectively; In addition, other methods have also played a certain role in improving the performance. Multi-task learning method has greatly improvement on SOC-150 dataset, increasing the recognition accuracy by 4.32%. Label Smoothing method and test time augmentation have also improved the performance to a certain extent, but have a relatively small impact.

In Table 10, the increased time cost of prototypical network compared with the time cose of the direct training method is 0.667 ms for each sample, and then the total time cost is almoste doubled. In particular, because the test time augmentation increases the single test sample to three test samples, the reasoning time reaches 2.968 ms. The addition of other methods has less impact on time cost.

**Figure 12:**
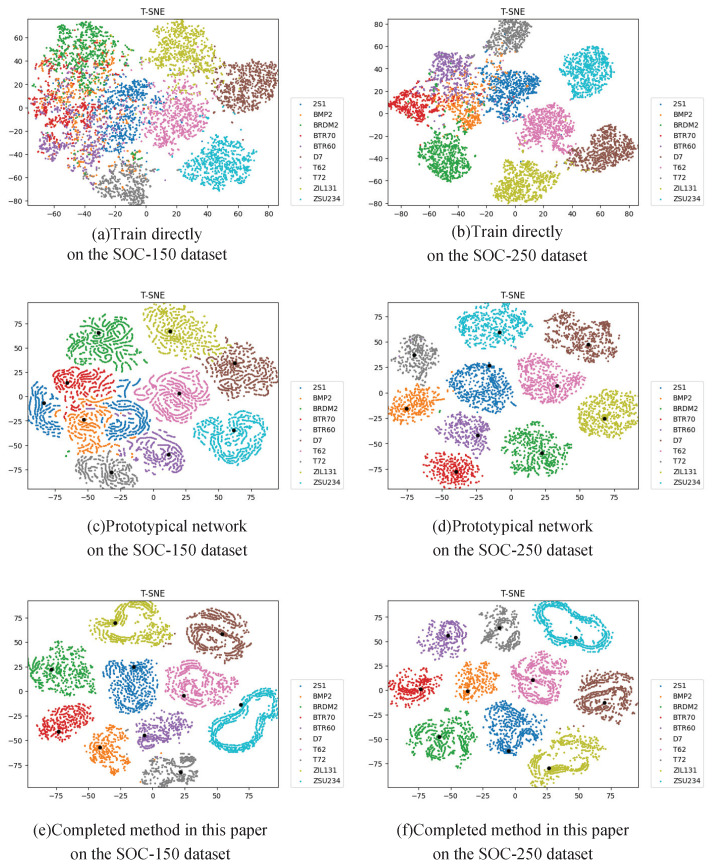
t-SNE visualization of testing dataset output.

In addition, we use t-SNE [32] to visualize the test set output of index 1, index 2, and index 7 in Table 9, correspond to the direct training method, the prototypical network, and the complete method proposed in this paper, respectively. As shown in Figure 12a,b, in the direct training method, the 2S1, BMP2, BTR70, and BTR60 are mixed and difficult to distinguish, which leads to low recognition accuracy. As shown in Figure 12c,d, the black dots are the prototypes calculated by the training set for each class. Compared with the direct training method, the output of the model trained by prototypical network is more tightly gathered to the prototypes. The output of SOC-250 dataset basically form an independent “island”, but there are still a few samples misclassified in SOC-150. As shown in Figure 12e,f, after applying the other methods and obtaining the complete method, the samples of each class are gathered more compactly in the prototype, and there are basically no test samples that have been classified incorrectly.

## 4. Discussion

### 4.1. Advantages

It can be seen from the experiments in Section 3 that compared with the direct training method, the method proposed in this paper can achieve higher recognition accuracy with a small number of training samples. The recognition performance has great improvement on a small number of training sample datasets of SOC1, EOC1 and EOC2, and slight improvement on a small number of training sample dataset of EOC3. This proves the advantages of the whole mothod in case of small datasets.

The ablation experiment in Section 3 verify the advantages of each part of the whole method. Among them, the prototypical network improves the recognition accuracy the most, the multi-task learning and the multi-level feature fusion methods improve the recognition accuracy secondly and thirdly. The NLECA module processes multi-level feature vectors and thus makes full use of the information of multi-aspect SAR images, which improves the recognition accuracy fourthly.

### 4.2. Generality

The experiments in Section 3 is conducted by using NLECA-EfficientNet as the feature extraction model. However, the method proposed in this paper has a certain degree of generality, and thus some other CNNs can be used as feature extraction models.

In order to prove the generality, this section uses three different CNNs: ResNet18 [24], VGG11 [25] and AlexNet [26] as feature extraction models to conduct experiments on the SOC-150 and SOC-250 datasets. The experimental results are shown in Table 11.

It can be seen from the experimental results that the method proposed in this paper can significantly improve the performance of target recognition when different CNNs are used as the feature extraction model, which proves that the method has a good generality.

## 5. Conclusions

Aimed at the problems of the multi-aspect SAR target recognition issue based on deep learning model under the small number of training samples, this paper proposes a small training dataset learning method based on the prototypical network. This method is classified by calculating the distance between the test sample and the prototype, and on this basis, methods such as multi-task learning, multi-level feature fusion and attention mechanism are added.

Experiments based on the MSTAR dataset show that this method can significantly improve the recognition performance of the deep learning model under a small number of samples, and thus the recognition accuracy can be close to that under the complete training set. In addition, the experiments also prove that this method has a certain generality and can be applied to a lot of deep learning feature extraction models. Therefore, this method is very meaningful and can be generally used for multi-aspect SAR target recognition issues with a small number of training samples.

Subsequent research can be carried out in the future. This method uses the amplitude SAR images for training and testing. The complex SAR images may make up for the lack of information in the case of a small number of training samples. Here, we refer to the CV-CNN method proposed in [33] to extend CNN to the complex domain, so as to make full use of SAR image information, and try to further improve the recognition accuracy under a small number of training samples.

## Figures and Tables

**Table 1:** EfficientNet-B0 network structure in this paper, MBConv is Mobile Inverted Bottleneck Convolution [23].

Stage	Operator	Channels	Layers
1	Conv3 × 3	32	1
2	MBConv1	16	1
3	MBConv6	24	2
4	MBConv6	40	2
5	MBConv6	80	3
6	MBConv6	112	3
7	MBConv6	192	4
8	MBConv6	320	1
9	Conv1d&Pooling&FC	*k*	1

**Table 2:** The size of SOC dataset.

Target	Train Dataset Size	Test Dataset Size
2S1	1162	1034
BMP2	883	634
BRDM_2	1158	1040
BTR70	889	649
BTR60	978	667
D7	1162	1037
T62	1162	1032
T72	874	642
ZIL131	1162	1034
ZSU_234	1162	1040
Total	10,592	8809

**Table 3:** The size of EOC1 dataset.

Target	Train Dataset Size	Test Dataset Size
2S1	1166	1122
BRDM_2	1162	1118
T72	913	1122
ZSU_234	1166	1122
Total	4407	4484

**Table 4:** Direct training parameter settings.

Type	Parameter
Batch Size	32
Optimizer	Adam
Adam’s Learning Rate	0.001
Center Loss’ Optimizer	SGD
SGD’s Learning Rate	0.5
Center Loss’ Hyper-Parameter	0.01
Cosine Decay Max Epoch	100
Epoch	100

**Table 5:** SOC datasets experimental results.

Dataset	Direct Training Acc.	Our Method Acc.	Increase in Acc.
SOC-150	77.06%	99.84%	21.34%
SOC-250	88.65%	99.97%	11.32%
Complete SOC Dataset	100%	-	-

**Table 6:** EOC1 datasets experimental results.

Dataset	Direct Training Acc.	Our Method Acc.	Increase in Acc.
EOC1-100	68.58%	98.38%	29.80%
EOC1-200	84.30%	99.26%	14.96%
Complete EOC1 Dataset	99.45%	-	-

**Table 7:** EOC2 datasets experimental results.

Dataset	Direct Training Acc.	Our Method Acc.	Increase in Acc.
EOC23-100	92.56%	99.39%	6.83%
EOC23-200	96.47%	99.67%	3.20%
Complete EOC23 Dataset	99.96%	-	-

**Table 8:** EOC3 datasets experimental results.

Dataset	Direct Training Acc.	Our Method Acc.	Increase in Acc.
EOC23-100	99.03%	99.28%	0.25%
EOC23-200	99.54%	99.69%	0.15%
Complete EOC23 Dataset	99.78%	-	-

**Table 9:** Recognition accuracy of ablation experiments.

	SOC-150 Dataset		SOC-250 Dataset	
Index	Accuracy	Increase in Accuracy	Accuracy	Increase in Accuracy
1	77.06%	-	88.65%	-%
2	86.30%	9.24%	95.45%	6.80%
3	90.62%	4.32%	96.34%	0.89%
4	94.74%	4.12%	98.49%	2.15%
5	97.60%	2.86%	99.42%	0.93%
6	97.91%	0.31%	99.56%	0.14%
7	98.40%	0.49%	99.97%	0.41%

**Table 10:** Time costs of ablation experiment.

Index	Time Costs	Increase in Time Costs
1	0.774 ms	-
2	1.441 ms	0.667 ms
3	1.519 ms	0.078 ms
4	1.602 ms	0.083 ms
5	1.702 ms	0.100 ms
6	1.739 ms	0.037 ms
7	2.968 ms	1.229 ms

**Table 11:** Experimental results under different CNN models.

Model	Dataset	Direct Training Acc.	Our Method Acc.	Increase in Acc.
ResNet18	SOC-150	87.10%	98.25%	11.15%
	SOC-250	92.85%	99.95%	7.10%
VggNet11	SOC-150	80.87%	99.13%	18.26%
	SOC-250	90.35%	99.80%	9.45%
AlexNet	SOC-150	85.52%	96.17%	10.65%
	SOC-250	94.15%	98.20%	4.05%

## Data Availability

Publicly available datasets were analyzed in this study. This data can be found here: [https://www.sdms.afrl.af.mil/] accessed on 24 April 2021.
